# Performance of New Roche Cobas Pulse Glucose Meter Against Potential Interfering Substances and Hematocrit Variations

**DOI:** 10.3390/diagnostics16091383

**Published:** 2026-05-01

**Authors:** Mokarrameh Pudineh Moarref, Wanda Black, Yu Chen

**Affiliations:** 1 Department of Laboratory Medicine, Dr. Everett Chalmers Regional Hospital, Horizon Health Network, Fredericton, NB E3B 5N5, Canada; mokarrameh.moarref@horizonnb.ca (M.P.M.); wanda.black@horizonnb.ca (W.B.); 2 Department of Pathology, Dalhousie University, Halifax, NS B3H 4R2, Canada; 3 Discipline of Laboratory Medicine, Memorial University of Newfoundland, St. John’s, NL A1B 3V6, Canada

**Keywords:** blood glucose monitoring, point-of-care testing, interference, Cobas Pulse, Accu-Chek Inform II

## Abstract

**Background**: Point-of-care (POC) glucometers are essential for rapid blood glucose monitoring but are subject to interference and hematocrit variations. This study evaluated the analytical performance of the new Cobas Pulse glucometer against the Accu-Chek Inform II meter in the presence of N-acetylcysteine (NAC, 0.32–2.5 mmol/L), ascorbic acid (0.28–2.84 mmol/L), D-galactose (5.5–27 mmol/L), hemolysis (0.5–5 g/L hemoglobin), icterus (200–1600 μmol/L bilirubin), lipemia (2.5–15 g/L Intralipid), and hematocrit variations (20–60%). **Methods**: Interference testing followed CLSI EP07 guidelines using three whole blood pools with low (2.0–2.7 mmol/L), medium (4.5–7.4 mmol/L), and high (16.3–23 mmol/L) glucose levels. Interferents were spiked into these whole blood pools. Duplicate glucose levels were measured by 2 Pulse meters and 2 Inform II meters. The results were then assessed using the international standards, e.g., ISO 15197:2017 criteria (±15% or ±0.83 mmol/L). **Results**: Accu-Chek Inform II showed severe positive interference from galactose (up to 446.3%, *p* < 0.001), ascorbic acid (up to 98.8%, *p* = 0.002), and NAC (up to 61.4%, *p* = 0.001), exceeding ISO limits. Cobas Pulse demonstrated minimal interference (maximum biases: −3.7% for galactose, −4.4% for ascorbic acid, 7.7% for NAC, all *p* > 0.05). Both meters showed similar hematocrit-dependent bias (positive at 20–30%, negative at 50–60%) and acceptable performance for hemolysis, icterus (≤800 μmol/L), and lipemia. **Conclusions**: Compared to the Accu-Chek Inform II, the Cobas Pulse demonstrated greater resilience to interferences. Cobas Pulse meets strict accuracy standards (±10% for hospital use) with low interference, which makes it suitable for care of critically ill patients. The Cobas Pulse is more dependable for POCT across various clinical situations, supporting its role in critical care.

## 1. Introduction

Rapid glycemic control in hospital settings, particularly in intensive care units (ICUs) and emergency departments, has become critically dependent on point-of-care (POC) glucose testing [1]. In critically ill patients, strict glycemic control is linked to better results; however, its effectiveness depends on the precision and consistency of glucose measurements [2,3]. Both endogenous and exogenous substances, as well as changes in sample matrix characteristics like hematocrit, can cause analytical interference with POC glucose meters [4,5]. These interferences may lead to clinically serious mistakes such as incorrect insulin dosage, postponed hypoglycemia treatment, or an inability to detect hyperglycemia [6].

High-dose intravenous antioxidants, such as ascorbic acid (vitamin C) and N-acetylcysteine (NAC), which are used in conditions like sepsis and acetaminophen overdose, are common interferents in the critical care setting [7,8]. Using specific enzyme systems, non-glucose sugars, like galactose, which are found in parenteral nutrition and in patients with inherited metabolic errors, may also result in false elevations in meters [9]. POC glucose accuracy is further complicated by sample abnormalities that are commonly seen in critically ill patients and patients in neonatal intensive care units, such as hemolysis, icterus, lipemia, and extreme hematocrit values [6,10].

Frameworks for methodically assessing these analytical interferences are provided by international standards, specifically the International Organization for Standardization (ISO) 15197 [11] and the Clinical and Laboratory Standards Institute (CLSI) guideline EP07 [12]. These guidelines establish acceptable limits for bias (e.g., ±15% or ±0.83 mmol/L) and define protocols for testing common interferents. Adherence to these standards is particularly critical for devices intended for use in high-acuity settings, where even small inaccuracies can have disproportionate clinical consequences.

The analytical performance of POC glucose meters is fundamentally determined by the enzyme system they employ. The glucose dehydrogenase-pyrroloquinoline quinone (GDH-PQQ) enzyme used in the Accu-Chek Inform II system is known to cross-react with non-glucose sugars (e.g., galactose, maltose, xylose) and can be directly oxidized by reducing agents such as NAC and ascorbic acid, leading to clinically significant positive bias [13]. This lack of specificity poses a particular risk in ICUs, where patients may receive concurrent therapies that introduce these interferents. In contrast, the more recent Cobas Pulse system employs a different enzymatic technology designed to mitigate such interference [14]. Specifically, it uses flavin adenine dinucleotide-dependent glucose dehydrogenase (FAD-GDH) on the working electrode to catalyze the oxidation of β-D-glucose. A key design feature is the incorporation of a redox mediator/electron acceptor that operates on both the working and reference electrodes at a low electrochemical potential. This low-potential design minimizes non-specific oxidation of interfering substances, ensures electron transfer proportional only to β-D-glucose concentration, and provides a built-in fail-safe error detection mechanism that flags or suppresses results when extreme interferent concentrations are present [15]. Understanding these mechanistic differences is essential for interpreting comparative performance data between devices.

To assess adherence to critical care standards, this study compares the Cobas Pulse and Accu-Chek Inform II for interference and hematocrit effects.

## 2. Materials and Methods

Testing for interference was conducted according to the guidelines set forth by CLSI EP07 [12]. Three fresh Li-heparin whole blood pools from apparently healthy outpatients were prepared daily with different glucose levels: low (2.0–2.7 mmol/L), medium (4.5–7.4 mmol/L), and high (16.3–23 mmol/L) by spiking D-Glucose (Cat# G8270) or diluting with normal saline. Samples were spiked with interferents N-acetylcysteine (NAC, Cat# A7250) 0.32–2.5 mmol/L; ascorbic acid (Cat# A92902) 0.28–2.84 mmol/L, D-galactose (Cat# G5388) 5.5–27 mmol/L; hemolysis 0.5–5 g/L hemoglobin using hemolysate; Intralipid (Cat# I141) 2.5–15 g/L; and bilirubin (Cat# B4126) 200–1600 µmol/L. All reagents were purchased from Milipore Sigma (Toronto, ON, Canada). Aliquots of hematocrit 20–60% were prepared by reconstituting packed red cells with autologous plasma of each Li-heparin whole blood pool. Testing was carried out in quadruplicate (n = 4) on 2 Cobas Pulse and 2 Accu-Chek Inform II meters (2 measurements each meter). Strips utilized during the study were Lot# 80306408 and Lot# 77278905 for Cobas Pulse and Lot# 671694 for Accu-Chek Inform II (provided by Roche Diagnostic, Laval, QC, Canada). The manufacturer had no role in study design, data collection, analysis, interpretation, or manuscript preparation.

Data was expressed as means and standard deviations (SD). Independent t-test was utilized to compare glucose results at different levels of interferents against baseline measurements (0 mmol/L or 0 g/L interferent) within the same glucose pool and meter type. A *p*-value < 0.05 was considered statistically significant. All statistical analyses were performed using Microsoft Excel (version 2402, Microsoft Corporation, Redmond, WA, USA).

The glucose changes in mmol/L (Δ) and the percentages of change (Δ%) from baseline were calculated. The application of error propagation in calculating the standard deviation of Δ was done by determining the square root of the sum of the two variance sources. The maximum bias for each interferent was identified as the largest absolute percent change (|Δ%|) from baseline across all tested concentrations within each glucose pool, separately for the Cobas Pulse and Accu-Chek Inform II meters. A measurement was considered to exceed the clinically acceptable limit if the bias surpassed the ISO 15197:2017 criteria (i.e., ±15% or ±0.83 mmol/L) [11]. This project was considered as part of a continuous laboratory quality improvement project that was approved by the Research Ethics Services of Horizon Health Network (Fredericton, NB, Canada). All volunteers provided written informed consent.

## 3. Results

### 3.1. Interference from N-Acetylcysteine (NAC)

**Figure 1:**
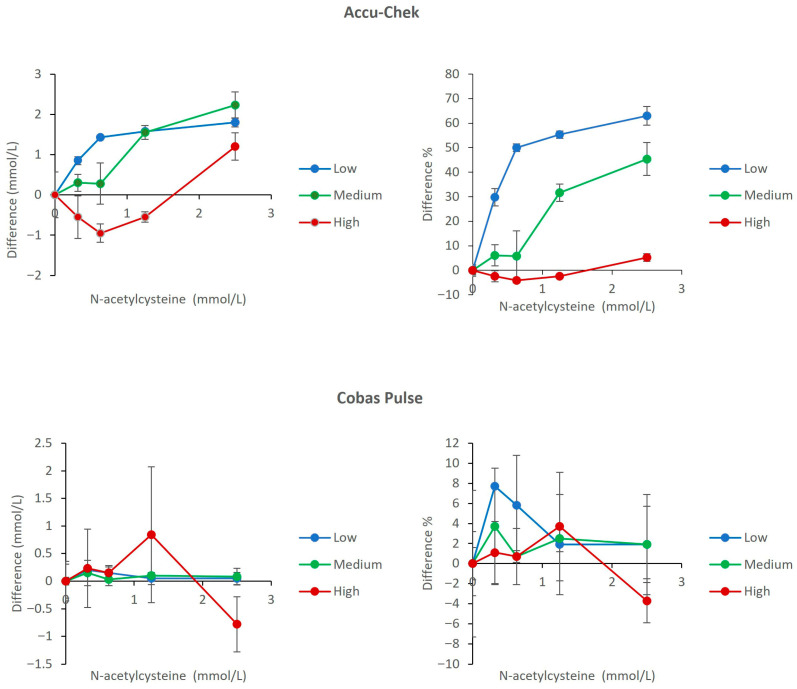
Effect of N-Acetylcysteine (NAC) on glucose measurements. The upper panels show results for the Accu-Chek Inform II meters (**left**: mean absolute bias in mmol/L; **right**: mean percent change relative to baseline). The lower panels show results for the Cobas Pulse meters (**left**: mean absolute bias in mmol/L; **right**: mean percent change relative to baseline). Data are plotted against NAC concentration for low (2.7 mmol/L), medium (4.5 mmol/L), and high (22 mmol/L) glucose pools. For each data point, mean bias (Δ) was calculated as the difference between the mean of four replicates at a given NAC concentration and the mean of four replicates at 0 mmol/L NAC (baseline) within the same glucose pool. Error bars represent the standard deviation of the difference.

The Accu-Chek Inform II demonstrated severe, dose-dependent positive interference from NAC, with the most pronounced effect in the hypoglycemic range (maximum bias: 61.4% at 2.85 mmol/L NAC). In contrast, the Cobas Pulse showed excellent resistance, with biases remaining within 7.7% across all NAC concentrations and glucose levels. Direct comparison of bias between the two systems visually underscores this dramatic difference (Figure 1).

### 3.2. Interference from Ascorbic Acid

**Figure 2:**
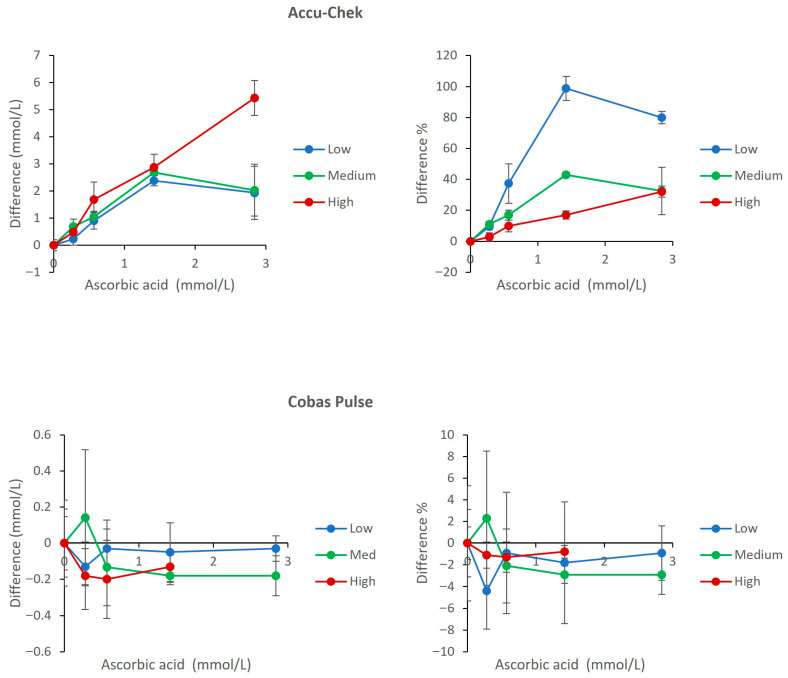
Effect of Ascorbic Acid on glucose measurements. The upper panels show results for the Accu-Chek Inform II meters (**left**: mean absolute bias in mmol/L; **right**: mean percent change relative to baseline). The lower panels show results for the Cobas Pulse meters (**left**: mean absolute bias in mmol/L; **right**: mean percent change relative to baseline). Data are plotted against ascorbic acid concentration for low (2.6 mmol/L), medium (5.9 mmol/L), and high (16.3 mmol/L) glucose pools. For each data point, mean bias (Δ) was calculated as the difference between the mean of four replicates at a given concentration and the mean of four replicates at 0 μmol/L ascorbic acid (baseline) within the same glucose pool. Error bars represent the standard deviation of the difference.

Ascorbic acid caused a profound positive interference on the Accu-Chek Inform II, with biases far exceeding ISO 15197 limits (up to 98.8% at 1.42 mmol/L in the low glucose pool. The Cobas Pulse exhibited only minimal negative bias (maximum −4.4%). Notably, at the highest concentration (2.84 mmol/L) in the hyperglycemic pool, the Cobas Pulse device triggered an error flag, effectively preventing erroneous reading, as shown in the comparative dose–response plot (Figure 2).

### 3.3. Interference from D-Galactose

**Figure 3:**
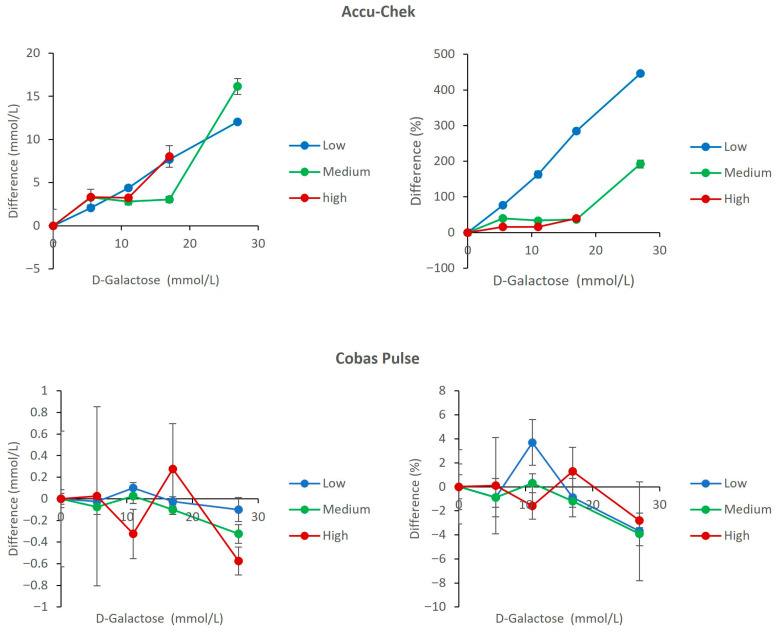
Effect of D-Galactose on glucose measurements. The upper panels show results for the Accu-Chek Inform II meters (**left**: mean absolute bias in mmol/L; **right**: mean percent change relative to baseline). The lower panels show results for the Cobas Pulse meters (**left**: mean absolute bias in mmol/L; **right**: mean percent change relative to baseline). Data are plotted against D-galactose concentration for low (2.7 mmol/L), medium (7.4 mmol/L), and high (19.2 mmol/L) glucose pools. For each data point, mean bias (Δ) was calculated as the difference between the mean of four replicates at a given concentration and the mean of four replicates at 0 mmol/L galactose (baseline) within the same glucose pool. Error bars represent the standard deviation of the difference.

The Accu-Chek Inform II exhibited severe positive interference in the presence of galactose, with a catastrophic bias exceeding 400% at a galactose concentration of 27 mmol/L in the low-glucose pool. At galactose concentrations of ≥17 mmol/L, glucose results were overestimated by more than 5 mmol/L across all glucose levels. Specifically, at 17 mmol/L galactose, positive biases of 7.68 mmol/L and 8.05 mmol/L were observed in the low- and high-glucose pools, respectively. At 27 mmol/L galactose, the magnitude of interference increased further, with overestimations reaching 12.05 mmol/L in the low-glucose pool and 16.15 mmol/L in the medium-glucose pool (Figure 3). In contrast, the Cobas Pulse demonstrated excellent resistance to galactose interference, with all measured biases remaining within −3.9% to 3.7% across all glucose and galactose concentrations tested (Figure 3).

### 3.4. Interference from Hematocrit Variations

**Figure 4:**
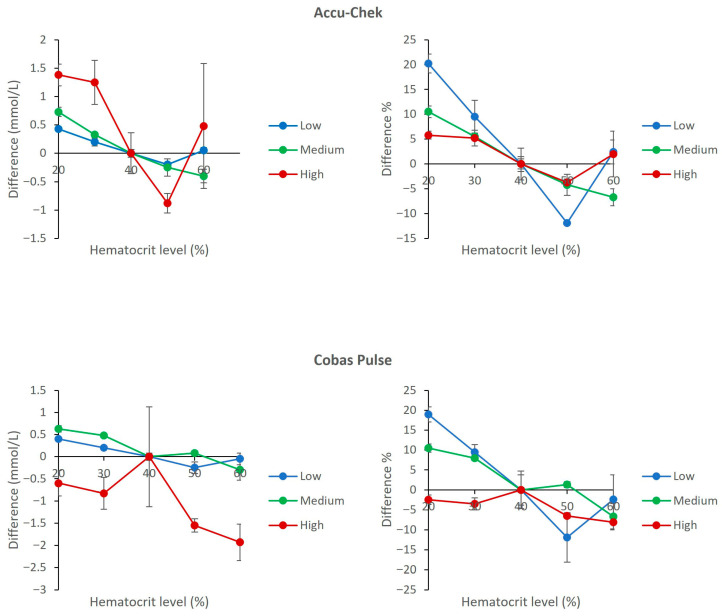
Effect of Hematocrit variation on glucose measurements. The upper panels show results for the Accu-Chek Inform II meters (**left**: mean absolute bias in mmol/L; **right**: mean percent change relative to reference). The lower panels show results for the Cobas Pulse meters (**left**: mean absolute bias in mmol/L; **right**: mean percent change relative to reference). Data are plotted against hematocrit levels for low (2.1 mmol/L), medium (5.4 mmol/L), and high (22.7 mmol/L) glucose pools. For each data point, mean bias (Δ) was calculated as the difference between the mean of four replicates at a test Hct and the mean of four replicates at 40% Hct (reference) within the same glucose pool. Error bars represent the standard deviation of the difference.

Both meters were comparably affected by deviations from the 40% hematocrit reference. The Accu-Chek Inform II showed a characteristic pattern of positive bias (dose-dependent) at low Hct and negative bias at high Hct. The Cobas Pulse exhibited a similar trend, as shown in its bias plots. The direct comparison illustrated that the maximum observed biases were approximately 20% at 20% Hct and approximately −8% at 60% Hct in the hypoglycemic pool (Figure 4).

### 3.5. Interference from Hemolysis

**Figure 5:**
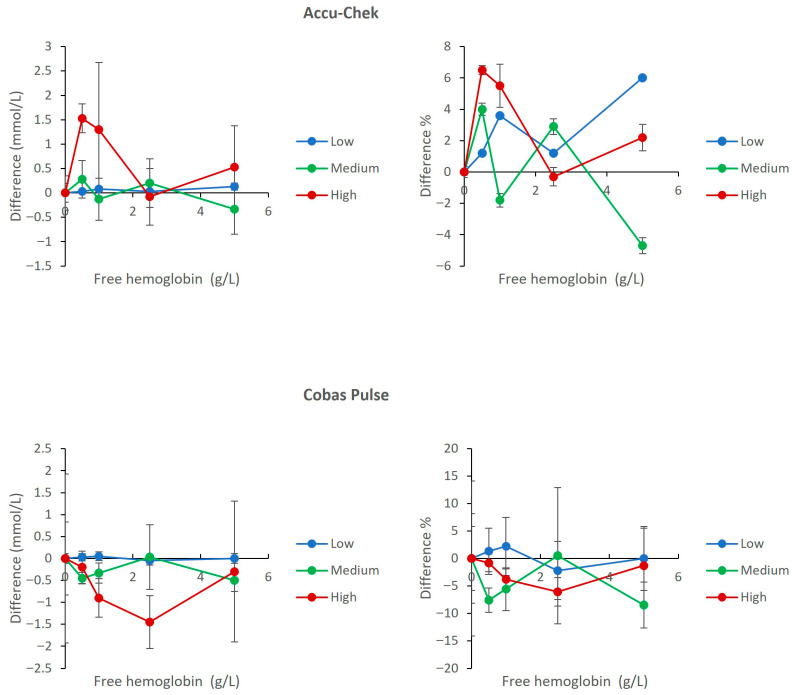
Effect of Hemolysis (free hemoglobin) on glucose measurements. The upper panels show results for the Accu-Chek Inform II meters (**left**: mean absolute bias in mmol/L; **right**: mean percent change relative to baseline). The lower panels show results for the Cobas Pulse meters (**left**: mean absolute bias in mmol/L; **right**: mean percent change relative to baseline). Data are plotted against hemolysate concentration for low (2.2 mmol/L), medium (6.9 mmol/L), and high (23 mmol/L) glucose pools. For each data point, mean bias (Δ) was calculated as the difference between the mean of four replicates at a given concentration and the mean of four replicates at 0 g/L hemoglobin (baseline) within the same glucose pool. Error bars represent the standard deviation of the difference.

Hemolysis up to 5 g/L hemoglobin induced only mild interference on both meters. The interference profile for Accu-Chek Inform II and Cobas Pulse demonstrated that maximum biases were within ±6.5% for Accu-Chek Inform II and within ±8.5% for Cobas Pulse. The comparative analysis across all pools confirmed both systems met ISO 15197 and CLSI EP07 acceptance criteria (Figure 5).

### 3.6. Interference from Icterus

**Figure 6:**
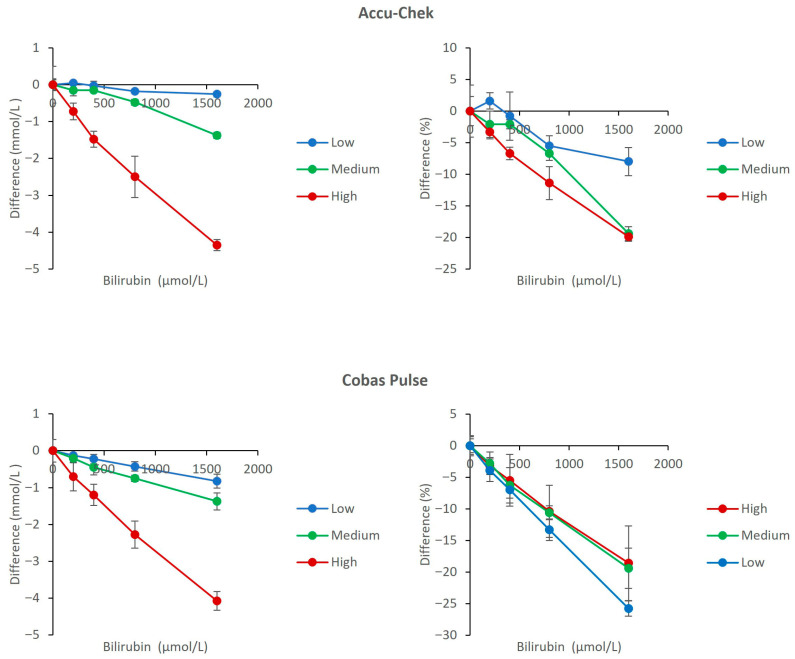
Effect of Bilirubin (icterus) on glucose measurements. The upper panels show results for the Accu-Chek Inform II meters (**left**: mean absolute bias in mmol/L; **right**: mean percent change relative to baseline). The lower panels show results for the Cobas Pulse meters (**left**: mean absolute bias in mmol/L; **right**: mean percent change relative to baseline). Data are plotted against bilirubin concentration for low (2.7 mmol/L), medium (6.5 mmol/L), and high (20.8 mmol/L) glucose pools. For each data point, mean bias (Δ) was calculated as the difference between the mean of four replicates at a given concentration and the mean of four replicates at 0 μmol/L bilirubin (baseline) within the same glucose pool. Error bars represent the standard deviation of the difference.

Elevated bilirubin in the vast majority of clinical ranges (up to 800 µmol/L) caused mild interference (within 15%) on both meters. The dose–response for Accu-Chek Inform II and Cobas Pulse showed a negative bias trend. The comparative analysis indicated maximum negative biases of −25.8% (Cobas Pulse, low glucose pool) and −19.9% (Accu-Chek Inform II, high glucose pool) at 1600 µmol/L (Figure 6).

### 3.7. Interference from Lipemia

**Figure 7:**
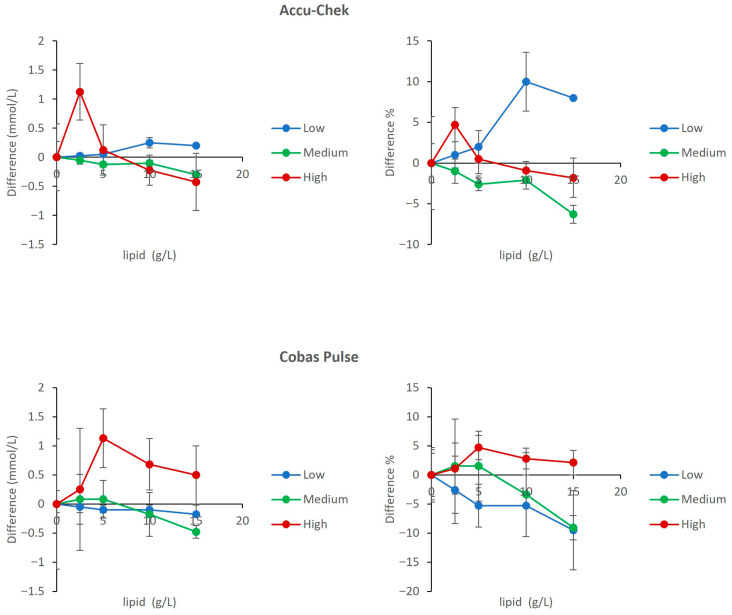
Effect of Lipid (lipemia) on glucose measurements. The upper panels show results for the Accu-Chek Inform II meters (**left**: mean absolute bias in mmol/L; **right**: mean percent change relative to baseline). The lower panels show results for the Cobas Pulse meters (**left**: mean absolute bias in mmol/L; **right**: mean percent change relative to baseline). Data are plotted against Lipid concentration for low (2.0 mmol/L), medium (5.0 mmol/L), and high (22 mmol/L) glucose pools. For each data point, mean bias (Δ) was calculated as the difference between the mean of four replicates at a given concentration and the mean of four replicates at 0 g/L Lipid (baseline) within the same glucose pool. Error bars represent the standard deviation of the difference.

Both devices demonstrated acceptable performance under lipemic conditions. The dose–response curves for Accu-Chek Inform II and Cobas Pulse showed mild interference. The direct comparison of absolute bias and percent bias across glucose pools confirmed that maximum biases were +10.0% (Accu-Chek Inform II) and −9.5% (Cobas Pulse), with corresponding absolute biases of approximately +0.35 mmol/L and −0.2 mmol/L, satisfying ISO 15197 criteria (Figure 7).

### 3.8. Summary of Maximum Bias and ISO 15197:2017 Compliance Across All Tested Interferents

To facilitate direct comparison of device performance across all tested conditions, Table 1 summarizes the maximum percent bias observed for Cobas Pulse and Accu-Chek Inform II meters for each interferent category, along with baseline glucose values (mean ± SD) and *p*-values from independent t-tests comparing maximum bias measurements to baseline (n = 4 per condition). The data demonstrate that Cobas Pulse remained within ISO 15197:2017 acceptance criteria (±15% or ±0.83 mmol/L) for all interferents at clinically relevant concentrations, whereas Accu-Chek Inform II exceeded these limits for NAC, ascorbic acid, and D-galactose (*p* < 0.01 for all three comparisons). Notably, Cobas Pulse also satisfied the stricter ±10% accuracy benchmark recommended for hospital use in critical care settings (Table 1).

For icterus, both meters were evaluated across the full range of 200–1600 μmol/L bilirubin. At the clinically important threshold of 800 μmol/L, neither device exceeded the ISO 15197:2017 limits of 15%. The differences from baseline were not statistically significant at this concentration (*p* > 0.05 for both meters). However, at the extreme concentration of 1600 μmol/L, both devices exceeded the ISO threshold, and the differences reached statistical significance (*p* < 0.05). Therefore, caution is warranted when interpreting POC glucose results in patients with severe hyperbilirubinemia (≥800 μmol/L), although routine clinical monitoring at bilirubin levels up to 800 μmol/L remains acceptable.

Hematocrit variations produced similar bias patterns on both meters. At low hematocrit (20%), both devices showed positive bias with maximum biases of 19.0% (Cobas Pulse) and 20.2% (Accu-Chek Inform II) in the low glucose pool, and the differences from baseline were statistically significant (*p* < 0.05 for both). At high hematocrit (60%), both devices showed negative bias, with Cobas Pulse showing −8.1% in the high glucose pool (*p* = 0.042) and Accu-Chek Inform II showing −6.7% in the medium glucose pool (*p* = 0.018). For hemolysis and lipemia, no statistically significant differences from baseline were observed (*p* > 0.05 for all concentrations).

## 4. Discussion

Using accurate point-of-care (POC) glucose measurements is a key part of care for critically ill patients [1]. Hyperglycemia and hypoglycemia each increase risks for complications and patient deaths [3,16]. The Intensive Care Unit (ICU) has many unique clinical and physiological difficulties that can affect how accurately a person’s glucose level is measured through POC devices. A major issue with accurate point-of-care glucose measurements within an ICU setting includes analytical interference from substances that can cause substantial clinical harm [17,18]. This study follows CLSI EP07 criteria and provides some of the objective evidence needed to help health care providers select devices when analytical reliability must be achieved. This study clearly identifies that the Cobas Pulse system has greater analytical reliability compared to the Accu-Chek Inform II for both the reducing agents, N-acetylcysteine (NAC) and ascorbic acid, as well as for galactose, which are all substances commonly found in an ICU setting.

### 4.1. Failure of Accu-Chek Inform II in Critical Care

Calibrating glucose meters for use in critically ill patients has several difficulties. These individuals often have rapidly changing body systems, multiple pre-existing medical conditions, and aggressive treatment plans that could lead to inaccurate glucose measurements [19,20]. Guidelines or consensus statements suggest that glucose meters used to monitor patients in a critical care setting must have both accuracy (defined as a measure of how correct an outcome can be) and a high level of analytical specificity; if this criterion is not met, it is possible that patients may experience clinically dangerous results due to inaccurate readings by the glucose meter [21,22].

As observed in our data, the Accu-Chek Inform II does not fulfill the necessary requirements for assay performance with several relevant interfering compounds such as NAC, ascorbic acid, and galactose. The amount of bias seen due to NAC (up to 61.4% in the low glucose pool) and ascorbic acid at 1.42 mmol/L (up to 98.8% in the low glucose pool) is far beyond the ISO 15197 limits; these biases could cause catastrophic clinical implications. For example, if a patient’s true glucose level was 2.5 mmol/L (hypoglycemic range), the value could be reported as high as 3.2–4.1 mmol/L in the presence of therapeutic NAC levels (0.32–0.63 mmol/L), and up to 4.1 mmol/L at 2.5 mmol/L NAC [23]. In the presence of ascorbic acid at 1.42 mmol/L, the reported glucose could reach 5.0 mmol/L, effectively masking hypoglycemia. With galactose, the overestimation is even more extreme, with values exceeding 13.5 mmol/L at galactose concentrations of 17–27 mmol/L (Figure 1, Figure 2 and Figure 3). The result is a potential failure to recognize and treat a life-threatening low blood sugar condition, or administration of an inappropriate amount of insulin; both scenarios represent an increased risk to patients [24,25].

### 4.2. Meeting the Stringent Requirements for Pulse Meters in Critical Care Use

In contrast, the performance of the Cobas Pulse consistently exceeded expectations, maintaining a level of accuracy well within the defined analytical limits (< ±10% bias and in many cases < ±5%) for all the critical interfering substances defined in our protocol (Figure 1, Figure 2 and Figure 3). The high degree of accuracy is likely due to the engineered enzyme/mediator system of the Cobas Pulse that makes it less likely to be oxidized by reducing agents and more specific for glucose [26]. An important safety feature is its ability to identify and reject samples with very elevated levels of ascorbic acid (2.84 mmol/L in high glucose pool) and withhold any result output for these samples (Figure 2). This fail-safe mechanism represents a major benefit to the ICU environment, whereby it will actively prevent a clinician from making a clinical judgement based on a markedly erroneous value.

To provide a structured, strategic overview of the comparative analytical and clinical attributes of the two POC glucose meters, a SWOT (Strengths, Weaknesses, Opportunities, Threats) analysis is presented in Table 2. This framework synthesizes the key experimental findings from the current study with contextual considerations for critical care implementation. Strengths and weaknesses are derived directly from the interference and hematocrit data presented in Figure 1, Figure 2, Figure 3, Figure 4, Figure 5, Figure 6 and Figure 7 and Table 1, while opportunities and threats incorporate broader clinical, operational, and safety-related factors relevant to ICU decision-making. The SWOT analysis is intended to assist laboratory directors, critical care physicians, and hospital administrators in making evidence-based decisions regarding POC glucose device selection (Table 2).

#### 4.2.1. The Impact of Pharmacological Interference: NAC and Ascorbic Acid

Some critically ill patients receive large amounts of reducing agents, like NAC (N-acetylcysteine), intravenously for treatment of acetaminophen poisoning and in conditions such as Acute Respiratory Distress Syndrome (ARDS) [27,28]. During standard intravenous NAC therapy for acetaminophen toxicity (300 mg/kg over 21 h), peak plasma NAC concentrations may reach approximately 0.3–0.6 mmol/L shortly after the loading infusion [28,29]. Although a healthy normal range circulating vitamin C level is only typically 50–70 µmol/L [30], extremely high-dose intravenous vitamin C, which has been increasingly investigated in sepsis and critically ill conditions, may produce pharmacologic plasma concentrations in the millimolar range depending on the administered dose and infusion rate [31]. For example, the average end-of-infusion peak plasma level after the last dose in 15 adult patients with advanced lung cancer given a 1.5 g/Kg dose by 2-h intravenous infusion three times weekly for 4 weeks reached 2.99 mmol/L [32]. The current study irrefutably demonstrates that the Accu-Chek Inform II GDH-PQQ enzyme system is extremely sensitive to both NAC and ascorbic acid and provides grossly inflated results; thus, the most dangerous and hazardous time to assess blood glucose levels using the device is when patients are hypoglycemic, leading to significant and catastrophic delays in treatment/management. Therefore, based on the current data, blood glucose measurement using the Accu-Chek Inform II for critically ill patients is likely to adversely affect the accuracy of blood glucose levels and consequently complicate glycemic control. Conversely, the Cobas Pulse demonstrates excellent performance in the presence of these interferents (bias < ±5%) with built-in error flags, positioning it as a reliable option for glucose monitoring in critically ill patient populations.

#### 4.2.2. The Galactose Threat in Parenteral and Metabolic Support

Galactose can be found in many types of parenteral nutrition formulations, and patients who have inherited metabolic disorders such as galactosemia may develop markedly elevated blood galactose concentrations, which can reach approximately 5–10 mmol/L in untreated or poorly controlled cases [33,34,35]. What is alarming about this is that the Accu-Chek Inform II reports with a huge positive bias (greater than 400%) when reacting to galactose as though it were glucose (Figure 3). Therefore, the Accu-Chek cannot be used for patients that are receiving parenteral nutrition or for infants that have galactosemia. The Cobas Pulse overcomes this issue by demonstrating a high level of specificity and a negligible bias of −3.7% to +3.7% (Figure 3), demonstrating that Cobas Pulse, like other advanced interference-resistant meters, is a reliable option for these clinical situations.

#### 4.2.3. Performance in Other Complex ICU Matrices

Figure 5, Figure 6 and Figure 7 show that both devices perform well and are comparable in the presence of other common ICU sample abnormalities including hemolysis, lipemia, and/or hyperbilirubinemia, within the ISO 15197 standards. Figure 4 demonstrates that both meters are affected by hematocrit (20% to 60%) in a predictable and similar way (positive bias with low Hct and negative bias with high Hct). The Cobas Pulse system has been validated across a wide hematocrit range. Goodman et al. evaluated contrived samples adjusted to hematocrit values of 40% to 65%, simulating the range observed in critically ill neonates, and found no endogenous interference from hematocrit on glucose measurements [36]. The bias values observed in our study across the tested hematocrit range (20–60%) mostly remained within the acceptable limits defined by both ISO 15197:2013 and the FDA’s professional BGMS guidance [37]. Nevertheless, it is imperative for clinicians working in an ICU to interpret glucose results (especially for hypoglycemia levels) with caution for patients with severe anemia or polycythemia, and this caution extends across all POC glucose monitoring devices [5,38].

#### 4.2.4. Summary of Interference Profiles and Evaluation Against Critical Care Requirements

To place the individual interference findings into a broader clinical decision-making context, the results were integrated and evaluated against the specific analytical performance criteria required for glucose meters used in intensive care unit (ICU) settings. These criteria developed from international standards measuring the accuracy (ISO 15197:2017) [11] and from the guidelines for point-of-care testing in hospital settings, primarily for testing the meter for resilience against common pharmacologic-induced (via drug interaction) and physiologic-induced (via changes in body function) interferents (occurring during testing) that are encountered with patient care delivery within the ICU [19,20,21].

Table 3 outlines the Pulse meter performance against the performance criteria for critical care application from ISO 15197:2017 limits, clinically acceptable bias, and clinical risk assessment. NAC, N-Acetylcysteine.

### 4.3. Limitations and Future Directions

Several methodological limitations should be considered when interpreting the findings of this study. First, all interference measurements were compared to baseline (neat) samples within the same glucose pool to isolate the effect of each interferent, which is consistent with CLSI EP07 guidelines [12].

However, this approach does not account for potential synergistic or antagonistic interactions between multiple concomitantly present interferents—a common scenario in critically ill patients receiving complex polypharmacy. Laboratory reference measurements using Cobas c702 (Roche Diagnostic, Laval, QC, Canada) and ABL835 (Radiometer Canada, Mississauga, ON, Canada) analyzers confirmed the true glucose concentrations in the tested samples, providing a robust benchmark for evaluating POC device performance. 

The experimental design relied on spiking experiments using blood from healthy outpatients rather than blood from actual critically ill patients. This in vitro model may not accurately replicate the in vivo matrix of ICU patients, which is characterized by dynamic fluctuations in metabolite levels, variable protein binding, altered pH, and the presence of multiple concurrent interferents. Consequently, the extent to which these findings correlate with real-world clinical scenarios remains uncertain.

Only two strip lots of the Cobas Pulse meter (Lot# 80306408 and 77278905) and one lot of the Accu-Chek Inform II (Lot# 67169485) were utilized. While this provides preliminary evidence of analytical performance, lot-to-lot and meter-to-meter variability should be systematically evaluated in future multi-site studies conducted in routine clinical settings.

In addition, limited statistical comparisons (e.g., independent t-test, mixed-effects models with device-by-interferent interaction terms) were performed between the two-meter types. Although the magnitude of bias differences is clinically striking—particularly for NAC, ascorbic acid, and galactose—inferential statistical testing with more replicate numbers would strengthen the generalizability of these conclusions and quantify the variance attributable to device, interferent concentration, and glucose level. Future studies should incorporate such analyses.

Finally, the sample pool was derived from a single geographic region and did not include diverse patient populations (e.g., neonates, geriatric patients, or individuals with hemoglobinopathies), which may affect the external validity of the findings.

Future directions should prioritize validation of these results using ex vivo samples obtained from critically ill patients actively receiving NAC therapy, high-dose ascorbic acid, or galactose-containing parenteral nutrition. Additionally, prospective observational studies comparing POC glucose readings with central laboratory reference methods in real-time ICU conditions would help determine whether the analytical advantages of Cobas Pulse translate into improved clinical outcomes, such as reduced hypoglycemic episodes or fewer insulin titration errors.

## 5. Conclusions

The analytical reliability of point-of-care (POC) glucose meters is vital to ensuring that patients in critical care settings do not suffer harm because of incorrect glucose readings. The results of this study show how critical it is to select a glucose meter (device) that is less likely to provide incorrect results because of interference.

Accu-Chek Inform II has marked susceptibility to the interference of N-acetylcysteine, ascorbic acid, and galactose. The use of this device in patient populations that receive any of these medications may create a serious risk of inaccurate glucose measurements and subsequent mismanagement of patient care. Cobas Pulse has excellent resistance to these 3 critical interferents while providing consistent performance across a wide variety of other laboratory conditions, such as abnormal samples with elevated serum indices and/or variations in hematocrit.

Therefore, hospitals and intensive care units that care for patients who are likely to receive large amounts of antioxidant therapy, be infused with N-acetylcysteine, or receive nutrition containing galactose should be using a Cobas Pulse or glucometers with similar performance as their preferred glucose monitoring device. This recommendation will support the overall objective of minimizing iatrogenic harm and maximizing glycemic control in patients who are at a greater risk of experiencing injury from incorrect glucose readings.

## Figures and Tables

**Table 1:** Summary of maximum biases (%) for Cobas Pulse and Accu-Chek Inform II across all tested interferents. Data are presented as mean ± SD. * *p* < 0.05 indicates statistically significant difference from baseline (n = 4 per condition). NAC, N-Acetylcysteine.

Interferent	Range	Cobas Pulse				Accu-Chek Inform II			
		Baseline Glucose (mmol/L)	Max Bias (%)	Glucose at Max Bias (mmol/L)	*p*-Value	Baseline Glucose (mmol/L)	Max Bias (%)	Glucose at Max Bias (mmol/L)	*p*-Value
NAC	0.32–2.5 mmol/L	2.60 ± 0.07	7.7	2.80 ± 0.11	0.312	2.85 ± 0.05	61.4	4.60 ± 0.11	0.001 *
		4.10 ± 0.31	2.0	4.18 ± 0.15	0.421	4.90 ± 0.07	38.8	6.80 ± 0.33	0.001 *
		21.10 ± 0.36	−3.8	20.30 ± 0.50	0.185	22.80 ± 0.57	8.3	24.70 ± 0.34	0.082
Ascorbic acid	0.28–2.84 mmol/L	2.80 ± 0.15	−4.4	2.70 ± 0.10	0.452	2.40 ± 0.04	98.8	4.50 ± 0.98	0.002 *
		5.60 ± 0.19	−2.9	5.40 ± 0.11	0.213	6.25 ± 0.11	43.0	8.20 ± 0.96	0.003 *
		15.50 ± 0.24	−0.8	15.40 ± 0.09	0.385	17.00 ± 0.21	32.0	22.40 ± 0.64	0.001 *
D-Galactose	5.5–27 mmol/L	2.85 ± 0.05	−3.7	2.75 ± 0.11	0.521	2.40 ± 0.12	446.3	14.50 ± 0.18	<0.001 *
		7.20 ± 0.08	−3.9	6.90 ± 0.09	0.184	7.50 ± 0.27	192.3	23.60 ± 0.93	<0.001 *
		19.40 ± 0.63	−2.8	18.90 ± 0.13	0.312	19.00 ± 1.91	39.1	27.00 ± 1.25	0.002 *
Hematocrit	20–60%	2.10 ± 0.04	19.0	2.50 ± 0.04	0.008 *	2.00 ± 0.04	20.2	2.40 ± 0.00	0.006 *
		4.90 ± 0.07	10.5	5.40 ± 0.05	0.007 *	5.80 ± 0.08	10.5	6.40 ± 0.15	0.012 *
		21.30 ± 0.29	−8.1	19.80 ± 0.41	0.018 *	24.10 ± 0.19	5.8	22.30 ± 0.17	0.008 *
Hemolysis	0.5–5 g/L	2.25 ± 0.11	2.2	2.35 ± 0.10	0.187	2.10 ± 0.04	6.0	2.23 ± 0.00	0.085
		6.80 ± 0.83	−8.5	6.90 ± 0.44	0.421	7.00 ± 0.19	−4.7	6.60 ± 0.52	0.213
		22.60 ± 1.92	−6.1	22.30 ± 1.60	0.385	23.50 ± 0.35	6.5	25.00 ± 0.84	0.084
Icterus	200–1600 μmol/L	2.85 ± 0.05	−25.8	2.00 ± 0.13	0.021 *	2.50 ± 0.13	−8.0	2.25 ± 0.07	0.034 *
		6.30 ± 0.08	−19.4	4.95 ± 0.23	0.003 *	6.60 ± 0.16	−19.4	5.20 ± 0.08	0.008 *
		20.50 ± 0.30	−18.6	16.40 ± 0.25	0.001 *	21.00 ± 0.50	−19.9	16.70 ± 0.15	0.002 *
Lipemia	2.5–15 g/L	2.00 ± 0.07	−9.5	1.80 ± 0.13	0.184	1.90 ± 0.00	10.0	2.25 ± 0.05	0.086
		4.60 ± 0.23	−9.1	4.10 ± 0.11	0.126	5.40 ± 0.27	−6.3	5.20 ± 0.05	0.213
		21.00 ± 1.12	2.1	21.50 ± 0.50	0.421	23.00 ± 0.57	−1.8	22.60 ± 0.49	0.312

**Table 2:** SWOT analysis of Cobas Pulse versus Accu-Chek Inform II for POC glucose monitoring in critical care. EMR, electronic medical record; FAD-GDH, flavin adenine dinucleotide-dependent glucose dehydrogenase; ICU, intensive care unit; NICU, neonatal intensive care unit; NAC, N-Acetylcysteine.

	Cobas Pulse	Accu-Chek Inform II
**Strengths**	FAD-GDH enzyme with high specificity; error flag at high ascorbic acid; meets ±10% hospital standard	Established track record; widely available; acceptable performance for routine samples without interferents
**Weaknesses**	Slight negative bias at high bilirubin (>800 μmol/L); limited long-term real-world ICU data	Severe positive interference with NAC, ascorbic acid, galactose; no fail-safe error flag
**Opportunities**	Adoption in ICUs, NICU, and metabolic units; integration with hospital EMR	May be repurposed for non-critical care settings where interferents are absent
**Threats**	Cost of transition; staff retraining; potential unknown interferents with FAD-GDH	Continued use in ICU despite evidence may lead to preventable patient harm

**Table 3:** Critical Care Glucometer Requirements vs. Device Performance.

Requirement for ICU Use	Cobas Pulse	Accu-Chek Inform II	Does Cobas Pulse Meet Requirement?
1. High Accuracy, especially in Hypoglycemia	Minimal bias across all glucose pools (max 7.7% in low pool) (Figure 1).	Severe positive bias in low glucose pool; up to 61.4% with NAC (Figure 1), 98.8% (Figure 2) with ascorbic acid	YES—Fully Meets. Pulse provides reliable measurements in the critical hypoglycemic range.
2. Resistance to Common ICU Drug Interferents (NAC, Ascorbic Acid)	Excellent resistance. Bias remained within ±5% at clinically relevant concentrations. An error flag at 2.84 mmol/L ascorbic acid prevented erroneous reporting (Figure 2).	Severe positive interference. Bias up to 98.8%, leading to falsely elevated readings and risk of untreated hypoglycemia (Figure 2).	YES—Fully Meets. Pulse is the safe choice for patients with these high-dose antioxidant therapies.
3. Specificity Against Non-Glucose Sugars (Galactose)	Excellent specificity. Negligible bias ( −3.7% to +3.7% %) (Figure 3) even at extreme concentrations (27 mmol/L) (Figure 3).	Catastrophic lack of specificity. Extreme positive bias (>400%) renders glucose readings meaningless (Figure 3).	YES—Fully Meets. Pulse is the only viable option in settings where galactose may be present.
4. Tolerance to Wide Hematocrit Variations (20–60%)	Acceptable performance. Exhibited the expected industry-standard pattern (positive bias at low Hct, negative at high Hct). All results within ISO 15197 limits (Figure 4).	Acceptable performance. Similar pattern and magnitude of bias, within ISO 15197 limits (Figure 4).	YES—Meets. (Requires clinical awareness for extreme values). Caution may be advised for interpreting hypoglycemia results.
5. Reliability in Lipemic and Hemolyzed Samples	Good performance. Bias < ±10% for lipids (Figure 7) and <±8.5% for hemolysis (Figure 5), within acceptable criteria	Good performance. Bias < ±10% for lipids (Figure 7) and <±8.5% for hemolysis (Figure 5), within acceptable criteria	YES—Meets. Both devices are reliable for these common sample issues.
6. Performance in Severe Hyperbilirubinemia	Mild to moderate negative bias observed. At concentrations ≤ 800 μmol/L, all results remained within ISO 15197 limits (±15%). At extreme concentrations (1600 μmol/L), some values exceeded the acceptable range (max −25.8%). (Figure 6).	Negative bias observed. At concentrations ≤ 800 μmol/L, all results remained within ISO 15197 limits. At extreme concentrations (1600 μmol/L), some values exceeded the acceptable range (max −19.9%). (Figure 6).	YES—Both devices meet ISO 15197 criteria for bilirubin concentrations ≤ 800 μmol/L. At extreme concentrations (1600 μmol/L), some values exceed the acceptable limits; therefore, caution is advised when interpreting results in patients with severe hyperbilirubinemia.
7. Error Detection and Fail-Safe Capability	PRESENT. At 2.84 mmol/L ascorbic acid in the high glucose pool, the device triggered a “Sample Unsuitable” error, suppressing a result (Figure 2).	ABSENT. Reported grossly inaccurate results even at extreme interferent concentrations.	YES—Fully Meets. Pulse incorporates an additional layer of patient safety.

## Data Availability

The data underlying this article are available in the article.
